# Inhibitory Effect of *Dodonaea viscosa* var. *angustifolia* on the Virulence Properties of the Oral Pathogens *Streptococcus mutans* and *Porphyromonas gingivalis*

**DOI:** 10.1155/2013/624089

**Published:** 2013-10-07

**Authors:** Mrudula Patel, Roxanne Naidoo, Foluso John Owotade

**Affiliations:** Division of Oral Microbiology, Department of Clinical Microbiology and Infectious Diseases, National Health Laboratory Services and Faculty of Health Sciences, University of the Witwatersrand, Private Bag 3, Wits, Gauteng, Johannesburg 2050, South Africa

## Abstract

*Aim.* This study investigated the effect of *Dodonaea viscosa* var. *angustifolia* (DVA) on the virulence properties of cariogenic *Streptococcus mutans* and *Porphyromonas gingivalis* implicated in periodontal diseases. *Methods. S. mutans* was cultured in tryptone broth containing a crude leaf extract of DVA for 16 hours, and the pH was measured after 10, 12, 14, and 16 h. Biofilms of *S. mutans* were grown on glass slides for 48 hours and exposed to plant extract for 30 minutes; the adherent cells were reincubated and the pH was measured at various time intervals. Minimum bactericidal concentration of the extracts against the four periodontal pathogens was determined. The effect of the subinhibitory concentration of plant extract on the production of proteinases by *P. gingivalis* was also evaluated. *Results.* DVA had no effect on acid production by *S. mutans* biofilms; however, it significantly inhibited acid production in planktonic cells. Periodontal pathogens were completely eliminated at low concentrations ranging from 0.09 to 0.02 mg/mL of crude plant extracts. At subinhibitory concentrations, DVA significantly reduced Arg-gingipain (24%) and Lys-gingipain (53%) production by *P. gingivalis* (*P* ≤ 0.01). *Conclusions.* These results suggest that DVA has the potential to be used to control oral infections including dental caries and periodontal diseases.

## 1. Introduction

The oral cavity is a complex ecosystem comprising many surfaces coated with a wide variety of species, and an opportunistic microflora which exists in the form of a biofilm may vary due to dietary constituents, systemic illnesses, poor saliva flow, and oral hygiene resulting in the alteration of the microbial communities which leads to the development of oral diseases.

*Streptococcus mutans *occurs in the oral cavity and has the ability to ferment dietary carbohydrates rapidly and produce acids which are responsible for the demineralization of enamel. The acidic environment also promotes the growth and virulence of the opportunistic pathogen *Candida* in the oral environment [1, 2]. In addition, it is implicated in root canal infections, odontogenic abscesses, and endocarditis. Soft tissue infections including periodontal disease are caused by obligate oral anaerobic gram negative bacteria, categorized as red and orange complexes of *Porphyromonas gingivalis*, *Fusobacterium nucleatum*, *Prevotella intermedia*, and others [3]. Among these opportunistic pathogens, *P. gingivalis* is the most aggressive organism mainly because it has the ability to produce proteases, for example, Arg-gingipain and Lys-gingipain as well as lipopolysaccharides, collagenases, and haemagglutinin and is protected by a capsule [4, 5]. In addition, *P. gingivalis* is linked to systemic diseases such as aortic atherosclerosis, myocarditis, myocardial infarction, and rheumatoid arthritis [6–8].

Oral hygiene products containing antimicrobial agents such as fluoride and chlorhexidine have been used to control biofilms containing *S. mutans* and *P. gingivalis*. In addition, the antimicrobial effect of many natural products has been tested on the virulence properties of *S. mutans,* that is, biofilm formation and acid production [9, 10].

Inhibitors of proteases produced by *P. gingivalis* are potentially new therapeutic agents that could control these organisms and related diseases [11]. *Dodonaea viscosa *var.* angustifolia *(DVA) also called hopbush, which is traditionally used for many ailments including oral infections [12, 13], is known to inhibit biofilm formation by *S. mutans* and reduce the virulence of *Candida albicans* [14, 15]. Furthermore, its cytotoxicity has also been established [16].

This study investigated the effect of a plant extract of the leaves of* Dodonaea viscosa *var.* angustifolia *on acid production by *S. mutans* and protease production by *P. gingivalis* and the antibacterial effect against *P. gingivalis*, *P. intermedia*, *F. nucleatum*, and *Capnocytophaga* species.

## 2. Materials and Methods

### 2.1. Cultures

Saliva and periodontal pocket debris samples from patients attending the Dental Clinic at Charlotte Maxeke Johannesburg Academic Hospital were collected and cultured on Mutans Bacitracin agar to isolate *Streptococcus mutans. *Blood agar supplemented with haem and menadione was used to isolate* P. gingivalis*, *Prevotella intermedia*, *Fusobacterium nucleatum*, and *Capnocytophaga *species. *S. mutans* cultures were identified using API 20 Strep auxanogram (bioMèrieux), and additional biochemical reactions. *S. mutans* NCTC 10919 and 4 clinical isolates of *S. mutans* were used in the study. *P. gingivalis*, *P. intermedia*, and* F. nucleatum* were identified using API 32A and PCR technique [17].* Capnocytophaga* spp. was identified using colony morphology and gram stain. Ethical clearance was obtained from The Committee for Research on Human Subjects (Medical), University of the Witwatersrand. Written consent was obtained from the subjects. Cultures were stored at −70°C until required. For each experiment, fresh inoculums containing approximately 10^6^ organisms per millilitre were prepared [14].

### 2.2. Plant Material and Extract Preparation

Plant material was collected from the Pipeklipberg, Mkhunyane Eco Reserve, Mpumalanga province of South Africa, verified (Voucher Specimens no. J 94882) as described previously [15].

Leaves of *Dodonaea viscosa *var.* angustifolia* were dried in the shade and milled to a fine powder. Thereafter, 1.0 g was extracted in 10 mL methanol with vigorous shaking and then centrifuged. The procedure was repeated three times [18]. The solvents were removed under a cold air stream, and a yield of 0.15 g dried extract was obtained. The crude dry extract was weighed and dissolved in DMSO to yield a solution containing 50 mg of crude extract per mL of DMSO. Similarly, extracts were prepared using acetone and ethanol as solvents. Fresh plant extracts were prepared for each experiment.

### 2.3. Minimum Bactericidal Concentration (MBC)

MBC tests were performed to determine the subinhibitory concentrations for the subsequent experiments. Twofold dilutions of crude plant extract were prepared in microtitre plates using appropriate medium; *P. gingivalis* and *P. intermedia,* Tryptone Soy broth containing haem and menadione; *Capnocytophaga *spp., Tryptone Soy broth; and *F. nucleatum,* Fusiform media. One hundred microlitres of each of the diluted plant extract was added to each of the 96-well round bottom microtitre plate. Fresh inocula with optical density of 0.2 (405 nm) containing approximately 10^5^-10^6^ organisms per millilitre were prepared, and each well was inoculated with 100 *μ*L of inocula. Microtitre plates containing *Capnocytophaga *spp. were incubated under CO_2_ at 37°C for 4 days. Plates containing *P. gingivalis*, *P. intermedia*, and *F. nucleatum* were incubated anaerobically at 37°C for 7 days. Chlorhexidine gluconate was used as a positive control and water as a negative control. Effect of DMSO was also measured. After incubation, each well was subcultured on blood agar. The lowest concentration that had no growth was recorded as MBC for that test organism. Each experiment was repeated in triplicate. Based on the MBC for *P. gingivalis*, subinhibitory concentrations were selected for the protease inhibition experiments. With *S. mutans*, the MBC of a previous study was used [14].

### 2.4. Acid Production by *S. mutans*

Methanol extract was used for the experiment. The effect of crude plant extract on the acid production by *S. mutans* grown in biofilms was studied using a technique described by Kim et al. [19], with modifications [19]. Fresh inocula of *S. mutans* with optical density of 0.2 (405 nm) containing approximately 10^5^-10^6^ organisms per millilitre were prepared.* S. mutans *biofilms were allowed to grow on glass slides in tryptone broth containing approximately 10^5^ cfu/mL *S. mutans* for 48 h with a change of the media after 24 h. Biofilms were then exposed to a 0.78 mg/mL subinhibitory concentration of crude plant extract [14] for 30 min and rinsed with phosphate-buffered saline (PBS). Thereafter, the adherent cells were transferred to 30 mL tryptone broth and incubated in CO_2_ at 37°C. The pH of the media was measured after 2, 4, 6, 8, 13, and 22 h. The effect of crude plant extract on the acid production by planktonic cells of* S. mutans *was tested by inoculating 30 mL of tryptone broth containing 0.78 mg/mL crude plant extract with 1 mL of inoculum containing 10^5^-10^6^ cfu/mL of *S. mutans* and incubated for 16 hours. The pH of the media was measured at 0, 10, 12, 14, and 16 h., and the *S. mutans* count was determined after 0, 12, and 16 hours using the microdilution technique [20].

In both of these experiments water instead of plant extract was used as a control. The effect of DMSO was also measured. Each experiment was repeated in triplicate for the 5 strains of *S. mutans* that were tested. The results were analysed using the Wilcoxon rank-sum test (Mann-Whitney).

### 2.5. Protease Inhibition

The inhibitory activity of the methanol plant extract against production of proteinases Arg-gingipain and Lys-gingipain by *P. gingivalis* was evaluated using the technique of Yamanaka et al. [21] with modifications [21].* P. gingivalis *was harvested by centrifugation at 5 000 g for 20 minutes, washed 3 times, resuspended in 50 mM phosphate-buffered saline (pH 7.4), and adjusted to an optical density of 2.0 at 660 nm. Benzoyl-arginine-*p*-nitroanilide and N-(p-Tosyl)-Gly-Pro-Lys 4-nitroanilide acetate salts in 0.1 M Tris-HCL (pH 8.0) containing 1 mM dithiothreitol were used as substrates for Arg-gingipain and Lys-gingipain, respectively.

One hundred microliters of the substrates was dispensed into the wells of a microtitre plate. Different concentrations of plant extract (including water as a control) and bacterial cell suspensions were added and incubated at 37°C for 20 min. Controls without substrate were included in each plate. Adsorption was measured at a wavelength of 405 nm. Relative enzymatic activity was determined as follows: [(A_405_ with bacterial cells and plant extract −A_405_ of control)/(A_405_ with bacterial cells −A_405_ of control)] × 100. Each experiment was repeated 10 times. The results were compared to the control, that is, water using Student's *t*-test.

## 3. Results

### 3.1. MBC

Periodontal pathogens were completely eliminated at low concentrations ranging from 0.09 to 0.02 mg/mL of crude plant extracts (Table 1). All the solvents gave similar results. Based on these results, subinhibitory concentrations (≤0.02 mg/mL) were selected for the virulence study of* P. gingivalis*. The antimicrobial effect of DMSO was lost at a concentration of 6.125% whereas chlorhexidine gluconate killed all the test organisms.

### 3.2. Acid Production

**Figure 1:**
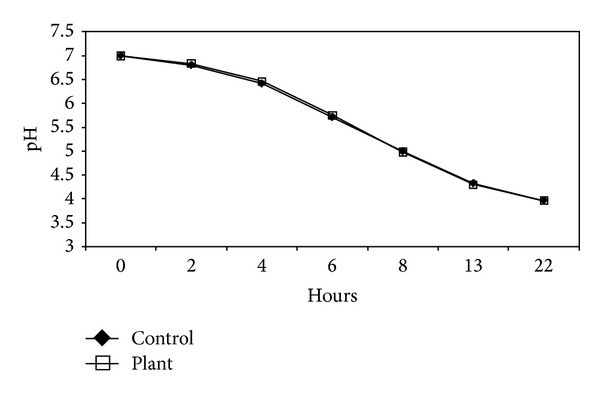
Effect of *Dodonaea viscosa *var.* angustifolia* on the acid production by biofilm of *S. mutans*.

**Figure 2:**
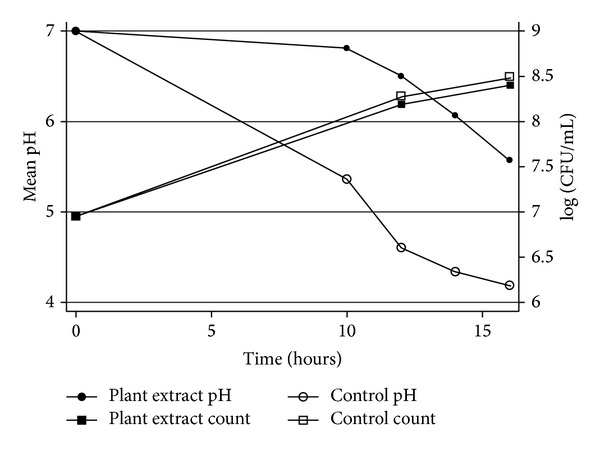
Effect of *Dodonaea viscosa *var.* angustifolia* on the acid production by planktonic *S. mutans*.

DVA had no effect on the acid production (*P* ≥ 05) by *S. mutans* biofilms (Figure 1); however, it significantly inhibited acid production (*P* ≤ 0.01) in planktonic cells (Figure 2). Bacterial counts in these cultures were performed to eliminate the possible antimicrobial effect of DVA at this subinhibitory concentration. The results showed that there was no significant difference in the bacterial counts of the control and the test cultures (*P* ≥ 0.05). This suggests that reduced acid production was due to the effect of DVA rather than the bacterial growth.

### 3.3. Proteinase Inhibition

**Figure 3:**
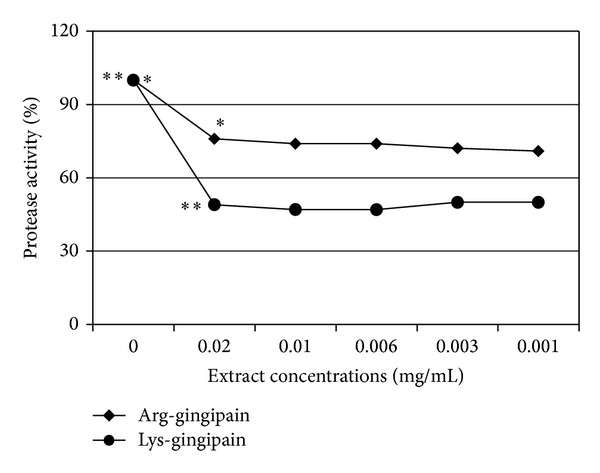
Effect of *Dodonaea viscosa *var.* angustifolia* on the production of Arg-gingipain and Lys-gingipain by *Porphyromonas gingivalis*. * and **: *P* ≤ 0.01.

DVA significantly (*P* ≤ 0.01) reduced the Arg-gingipain and Lys-gingipain production by *P. gingivalis* (Figure 3). The highest reduction of Arg-gingipain was at 0.02 mg/mL DVA (24%) whereas 53% reduction in the production of Lys-gingipain was achieved at 0.01 mg/mL.

## 4. Discussion 

In the last decade due to the escalation in the development of drug resistance in microorganisms, a novel approach has been proposed whereby the therapeutic agent can target the virulence of causative organisms rather than the number of infectious agents [22, 23]. This approach can be applied to commensals that cause opportunistic infections as they are often not eliminated completely.

This study showed that a subinhibitory concentration of the crude extract of *D. viscosa *var.* angustifolia* significantly inhibits acid production by *S. mutans*, which is an important virulence factor of this organism. *S. mutans* ferments dietary carbohydrates and produces acids which can cause demineralization of teeth [24]. As demonstrated in this study, the presence of plant extract will not allow *S. mutans* to produce acid, thereby preventing dental caries. This effect was not dependent on the number of *S. mutans* because the cfu/mL of *S. mutans* was not significantly different from the controls. This suggests that the phosphotransferase (PTS) uptake system that assists the transport of glucose could not have been affected by the plant extract. However, conversion of glucose to pyruvate by EMP pathway may have been affected. Polyphenols and tannins are known to interact with cellular enzymes that are responsible for the metabolic pathways, and these compounds have been identified from DVA [14, 25–27].

Our results also showed that acid production by *S. mutans* in the biofilm form is not affected by DVA, though it is known to prevent biofilm formation [14]. If DVA is added to oral hygiene products, regular use will inhibit the plaque formation as well as acid production by planktonic cells of *S. mutans*. The unfavorable results regarding acid production by the biofilms may have been due to the extracellular polysaccharides that are normally produced in the biofilm by *S. mutans.* They are tenacious and may have prevented the penetration of many large molecules including chemicals present in the plant extract.

DVA inhibited the growth of some of the anaerobic gram negative bacteria including *P. gingivalis* that are implicated in soft tissue infections. In addition, it reduced the production of Arg-gingipain by 24% and Lys-gingipain by 53%, which are the most important virulence factors of *P. gingivalis*. These proteinases downregulate polymorphonuclear neutrophils, modulate host cytokine networks, and degrade proteins which are present in the gingival tissues [21, 28]. Gingipains are essential to *P. gingivalis *as they provide iron, peptides, and amino acids from environmental proteins [29]. In addition, they are responsible for the cleavage of human transferrin which promotes growth and formation of hydroxyl radicals that play an important role in tissue destruction during infection [30]. Therefore, inhibition of gingipains by DVA will also deprive *P. gingivalis* of available iron and prevent some tissue destruction. It has been suggested that polyphenols and catechins may be responsible for the antibacterial and antiproteolytic effects against *P. gingivalis *[21, 30, 31] which are also present in DVA [14].

The results of this study have shown that if DVA is incorporated into a mouth rinse, gel or toothpaste at high concentrations, it will eliminate four of the major periodontal pathogens including *P. gingivalis. *Even though the concentration of the plant extract is reduced in the oral cavity due to salivary flow, it will render *S. mutans* and *P. gingivalis* avirulent. However, further research is needed to identify the active ingredient responsible for the beneficial effects.

## 5. Conclusions

At subinhibitory concentrations a crude extract of DVA renders *S. mutans* and *P. gingivalis *avirulent by preventing acid and proteinase production, respectively. In addition, it kills *P. gingivalis*, *P. intermedia*, *F. nucleatum*, and *Capnocytophaga *species at low concentrations. This supports the suggestion that the extract of this plant has the potential to be used as preventive and therapeutic agent for the treatment of infections of the mouth.

## Figures and Tables

**Table 1:** Minimum bactericidal concentrations of *Dodonaea viscosa *var.* angustifolia *against periodontal pathogens.

Cultures	Median MBC in mg/mL (*n* = 3)		
	Acetone	Ethanol	Methanol
*P. intermedia *	0.04	0.09	0.04
*P. gingivalis *	0.04	0.09	0.09
*F. nucleatum *	0.09	0.09	0.04
*Capnocytophaga *	0.04	0.02	0.02

Lowest MBC of DMSO was 6.125% and chlorhexidine gluconate killed all the test organisms.

## References

[1] Samaranayake LP, MacFarlane TW (1982). Factors affecting the in vitro adherence of the fungal oral pathogen *Candida albicans* to epithelial cells of human origin. *Archives of Oral Biology*.

[2] Chopde N, Jawale B, Pharande B, Chaudhari L, Hiremath V, Redasani R (2012). Microbial colonization and their relation with potential cofactors in patients with denture stomatitis.. *Journal of Contemporary Dental Practice*.

[3] Haffajee AD, Socransky SS, Patel MR, Song X (2008). Microbial complexes in supragingival plaque. *Oral Microbiology and Immunology*.

[4] Eley BM, Cox SW (2003). Proteolytic and hydrolytic enzymes from putative periodontal pathogens: characterization, molecular genetics, effects on host defenses and tissues and detection in gingival crevice fluid. *Periodontology 2000*.

[5] Darveau RP, Hajishengallis G, Curtis MA (2012). *Porphyromonas gingivalis* as a potential community activist for disease. *Journal of Dental Research*.

[6] Rivera MF, Lee JY, Aneja M, Goswami V, Liu L, Velsko IM (2013). Polymicrobial with major periodontal pathogens induced periodontal disease and aortic atherosclerosis in hyperlipidemic ApoE(null) mice.. *PLoS One*.

[7] Akamatsu Y, Yamamoto T, Yamamoto K (2011). *Porphyromonas gingivalis* induces myocarditis and/or myocardial infarction in mice and IL-17A is involved in pathogenesis of these diseases. *Archives of Oral Biology*.

[8] Bingham CO, Moni M (2013). Periodontal disease and rhenmatoid arthritis: the evidence accumulates for complex pathobiologic interactions. *Current Opinions in Rheumatology*.

[9] Koo H, Gomes BPFA, Rosalen PL, Ambrosano GMB, Park YK, Cury JA (2000). In vitro antimicrobial activity of propolis and *Arnica montana* against oral pathogens. *Archives of Oral Biology*.

[10] Xiao J, Zhou XD, Feng J, Hao YQ, Li JY (2007). Activity of nidus vespae extract and chemical fractions against *Streptococcus mutans* biofilms. *Letters in Applied Microbiology*.

[11] Grenier D, La VD (2011). Proteases of porphyromonas gingivalis as important virulence factors in periodontal disease and potential targets for plant-derived compounds: a review article. *Current Drug Targets*.

[12] Qureshi SJ, Khan MA, Ahmad M (2008). A survey of useful medicinal plants of Abbottabad in northern Pakistan. *Trakia Journal of Sciences*.

[13] Cribb AB, Cribb JW (1981). *Wild Medicine in Australia*.

[14] Naidoo R, Gulube Z, Fenyvesi I, Patel M (2012). Inhibitory activity of *Dodonaea viscosa var. angustifolia* extract against *Streptococcus mutans* and its biofilm. *Journal of Ethnopharmacology*.

[15] Patel M, Gulube Z, Dutton M (2009). The effect of *Dodonaea viscosa var. angustifolia* on *Candida albicans* proteinase and phospholipase production and adherence to oral epithelial cells. *Journal of Ethnopharmacology*.

[16] Khalil NM, Sperotto JS, Manfron MP (2006). Antiinflammatory activity and acute toxicity of dodonaea viscosa. *Fitoterapia*.

[17] Patel M, Coogan M, Galpin JS (2003). Periodontal pathogens in subgingival plaque of HIV-positive subjects with chronic periodontitis. *Oral Microbiology and Immunology*.

[18] Eloff JN (1999). The antibacterial activity of 27 southern African members of the combretaceae. *South African Journal of Science*.

[19] Kim J-E, Kim H-E, Hwang J-K, Lee H-J, Kwon H-K, Kim B-I (2008). Antibacterial characteristics of curcuma xanthorrhiza extract on *Streptococcus mutans* biofilm. *Journal of Microbiology*.

[20] Kramer J (1977). A rapid microdilution technique for counting viable bacteria in food. *Laboratory Practice*.

[21] Yamanaka A, Kouchi T, Kasai K, Kato T, Ishihara K, Okuda K (2007). Inhibitory effect of cranberry polyphenol on biofilm formation and cysteine proteases of *Porphyromonas gingivalis*. *Journal of Periodontal Research*.

[22] Alksne LE, Projan SJ (2000). Bacterial virulence as a target for antimicrobial chemotherapy. *Current Opinion in Biotechnology*.

[23] Escaich S (2008). Antivirulence as a new antibacterial approach for chemotherapy. *Current Opinion in Chemical Biology*.

[24] Nisengard RJ, Newman MG (1994). *Oral Microbiology and Immunology*.

[25] Duarte S, Gregoire S, Singh AP (2006). Inhibitory effects of cranberry polyphenols on formation and acidogenicity of *Streptococcus mutans* biofilms. *FEMS Microbiology Letters*.

[26] Leitão DPDS, Da Silva Filho AA, Polizello ACM, Bastos JK, Spadaro ACC (2004). Comparative evaluation of *in vitro* effects of Brazilian green propolis and baccharis dracunculifolia extracts on cariogenic factors of *Streptococcus mutans*. *Biological and Pharmaceutical Bulletin*.

[27] Maeyama R, Kwon IK, Mizunoe Y, Anderson JM, Tanaka M, Matsuda T (2005). Novel bactericidal surface: catechin-loaded surface-erodible polymer prevents biofilm formation. *Journal of Biomedical Materials Research A*.

[28] Brochu V, Grenier D, Nakayama K, Mayrand D (2001). Acquisition of iron from human transferrin by *Porphyromonas gingivalis* a role for Arg- and Lys-gingipain activities. *Oral Microbiology and Immunology*.

[29] Grenier D, Imbeault S, Plamondon P, Grenier G, Nakayama K, Mayrand D (2001). Role of gingipains in growth of *Porphyromonas gingivalis* in the presence of human serum albumin. *Infection and Immunity*.

[30] Bodet CC, Piché M, Chandad F, Grenier D (2006). Inhibition of periodontopathogen-derived proteolytic enzymes by a high-molecular-weight fraction isolated from cranberry. *Journal of Antimicrobial Chemotherapy*.

[31] Okamoto M, Sugimoto A, Leung K-P, Nakayama K, Kamaguchi A, Maeda N (2004). Inhibitory effect of green tea catechins on cysteine proteinases in *Porphyromonas gingivalis*. *Oral Microbiology and Immunology*.

